# Root of a Canine Tooth Lodged in the Thickness of the Lower Lip, Simulating a Cancerous Tumor

**Published:** 1862-08

**Authors:** 


ROOT OF A CANINE TOOTH LODGED IN THE THICKNESS OF THE LOWER LIP, SIMULATING A CANCEROUS TUMOR.—M. Landyck, of Dunkirk, has published this case, referring to a lady forty years of age. She had always suffered with her teeth, and had but a few incisors left, the rest of the jaws presenting roots more or less firmly wedged, and alveoli more or less decayed. Towards the end of the year 1854, she felt a small tumor forming in the lower lip on the left side, which tumor soon filled the space below the internal aspect of the lip and gum. Pain was subsequently experienced, and a few months afterwards the patient could hardly eat. She sent for M. Landyck, who discovered a large tumor situated as above described, and ulcerating. Ablation was not proposed, as the lady was very nervous, and cauterization was resorted to. In the meanwhile parturision took place quite normally, and, while using the caustic some time afterwards, M. Landyck felt a hard substance in the tumor ; by then making a crucial incision, he discovered a long root of the canine tooth, covered with a thick layer of calcareous matter. It was placed horizontally, the apex turned towards the lip, and its upper part adhering to the bone. This being removed, the tumor disappeared and the patient was freed from all uneasiness.—*Lancet.*

